# Antegrade nailing evolution for proximal humeral fractures, the Telegraph IV^®^: a study of 67 patients

**DOI:** 10.1007/s00590-014-1493-1

**Published:** 2014-06-20

**Authors:** Christian Cuny, Thomas Goetzmann, Delphine Dedome, Jean-Baptiste Gross, M’Barek Irrazi, Aboubekr Berrichi, Julien Mayer, Pierre-Yves Le Coadou, Sorin Precup, Laurent Galois, Didier Mainard

**Affiliations:** 1 Department of Orthopedics and Traumatology, Metz Mercy Regional Hospital, CHR Metz Mercy, CS45001, 57085 Metz Cedex 03, France; 2 Department of Orthopedic Surgery and Traumatology, Nancy Central University Hospital, Nancy, France

**Keywords:** Proximal humeral fractures, Antegrade nailing, Locking screws

## Abstract

**Introduction:**

There are multiple surgical treatment methods for proximal humerus fractures (PHF), but rarely do they provide satisfactory results. The objective of this study was to assess radioclinical outcomes and complications in patients treated using a modern intramedullary nailing system the Telegraph IV^®^.

**Materials and methods:**

This is an observational multicenter study cohort conducted between March 2008 and December 2009 on 105 patients admitted with a diagnosis of PHF and operated on two trauma I centers. The Neer and Articular Surgical neck Tuberosities classifications were used for the study. The primary outcome measure was the clinical Constant score. Follow-up of the patients was done at 6 weeks, 3 months, 6 months, 1 year, and 3 years after the procedure.

**Results:**

A total of 67 patients (51 women and 16 men) were assessed at a mean of 38 months. The weighted Constant score was 88 %. The mean rate of complications was 16 %. The weighted Constant scores were 84 and 95 % for the 2- and 3-part groups, respectively. Articular 4-part fractures had an average score of 86 % when they were valgus impacted and 67 % for complex disengaged fractures. Notably, the complication rate was 67 % for this latter group.

**Conclusions:**

Our clinical results support the use of this antegrade nailing for extra-articular and valgus-impacted articular fractures. This procedure does not appear suitable for displaced articular fracture for which arthroplasty may be indicated by elderly.

## Introduction

The incidence of proximal humeral fractures is increasing and accounts for 5.7 % of the current orthopedic trauma volume, according to Court-Brown [1]. This increase is especially true in the elderly osteoporotic population [1–3]. Nevertheless, the management of proximal humerus fractures remains controversial, especially for complex fractures, which are burdened with a high rate of complications and poor functional outcomes. Treatment options range from nonsurgical management to intramedullary nailing, open reduction internal fixation or arthroplasty. There appears to be significant geographical variation in regard to the treatment modalities employed for these injuries as well as a lack of evidence-based practice [4]. Many orthopedic surgeons choose to use intramedullary nailing when the fracture is amenable to fixation, while other orthopedic traumatologists tend to favor the use of a locking plate. Notably, the Telegraph IV^®^ nail (FH Orthopedics, Heimsbrunn, France) [5, 6] was designed to improve biomechanical stability of the construct, using a more modern system with four proximal locked in the nail screws. However, this system remains to be carefully evaluated for the treatment of proximal humerus fractures. Therefore, the objective of the present study was to assess the midterm radioclinical results and complications associated with a consecutive series of proximal humeral fractures treated with this contemporary intramedullary nailing system. Taken together, our findings contribute to a better understanding of the safety and efficacy of this therapeutic procedure.

## Materials and methods

### Patients

This is an observational, multicenter study cohort conducted on 105 patients admitted with a diagnosis of proximal humerus fractures and operated on between March 2008 and December 2009 at two level I centers. Approval from the local ethics committee and the Institutional Review Board (IRB) was granted (number: 2013-BCE-06). All patients gave written informed consent prior to study participation.

Patients presenting at our level I trauma centers with a proximal humerus fracture, for which a collective decision of surgical treatment by osteosynthesis was taken, were included in the study. The following exclusion criteria were applied: age <18 years old, unable to give informed consent, proximal humerus fracture associated with other orthopedic injuries and open fractures.

### Surgery and follow-up

**Fig. 1:**
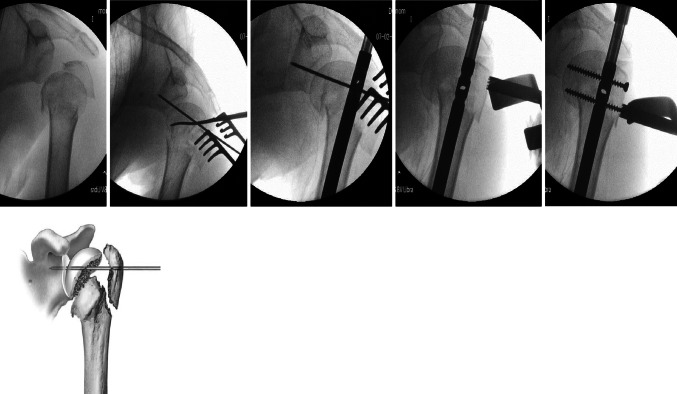
Crucifixion technique in which the head is fixed on the glenoid with a K-wire after reduction

The average time from injury to surgery was 2 days (0–26 days). Patients were placed in a beach chair position with the shoulder protruding from the table and the arm extended. The image intensifier was placed perpendicular to the arm’s axis. Percutaneous access (PC) in front of the acromion was carried out in 56 cases (53 %), whereas the remaining patients underwent anterolateral (AL) access. In all cases, the cephalic entry point of the Telegraph 4 nail was performed at the top of the humeral head, in an area of joint cartilage, through the muscular section of the rotator cuff. Bone sutures were used in 19 cases (18 %), and the crucifixion (Fig. 1) technique employed in 22 cases (21 %) to ensure the best possible reduction in the fracture (Table 2). The assembly was quick for all patients. Notably, the crucifixion (Fig. 1) consisted of raising the humeral head with a spatula until the anatomical reduction could be temporarily stabilized by a K-wire between the head (i.e., the posterior part in order to avoid contact when the nail goes down) and the glenoid. The tuberosities, which were reduced with a hook or forceps, were stably attached to the nail using screws or bone sutures.

The need for postoperative immobilization was determined by the surgeon according to bone quality, intraoperative stability of the fixation, and the type of fracture. Forty percent of patients were not immobilized, while the remaining subjects were immobilized for an average of 3 weeks using a splint sling. Physiotherapy began 3 weeks postoperatively for all patients. Patients were reviewed at 6 weeks and 3 months postoperatively by their surgeons. Physiotherapy was initiated from the time of immobilization removal and active mobilization began at 6 weeks.

Follow-up was conducted at 2 weeks, 6 weeks, 3 months, 6 months, 1 year, and 3 years post procedure. At each follow-up visit, patients were seen by the research coordinator along with either residents or their attending surgeon. The primary outcome measure was the clinical Constant score, as determined by three resident orthopedic surgeons between November 2011 and April 2012 with a mean follow-up of 32 months post surgery (range 19–47 months). Strength was assessed using a spring balance. Notably, all discrepancies were examined by a staff committee. Secondary outcome measures included radiological analysis of the fracture reduction and the occurrence of adverse events, such as infections, osteonecrosis, construct failure, secondary displacement, algodystrophy, and reoperation.

### Assessment methods

**Fig. 2:**
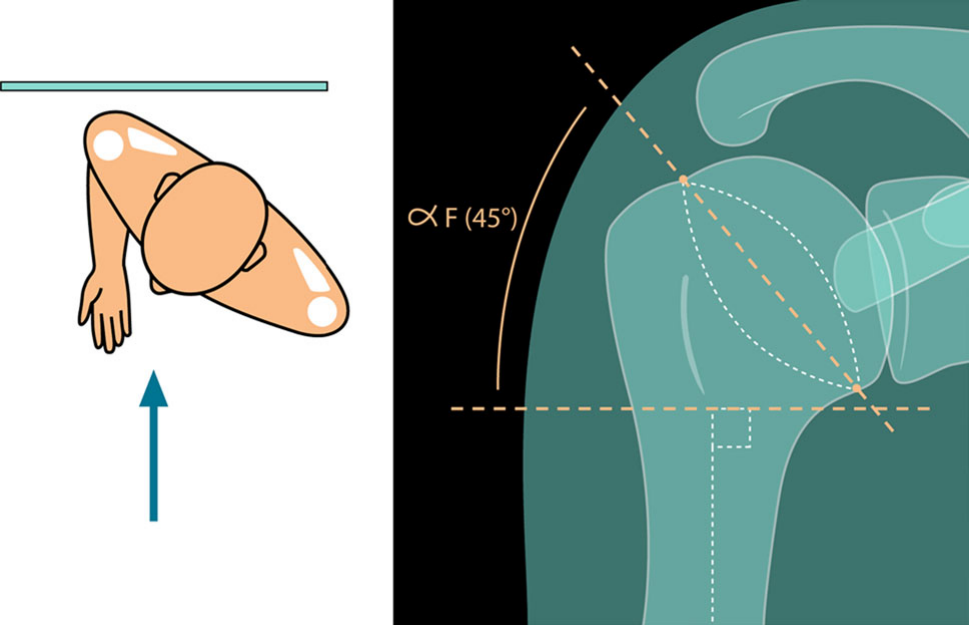
AP view

**Fig. 3:**
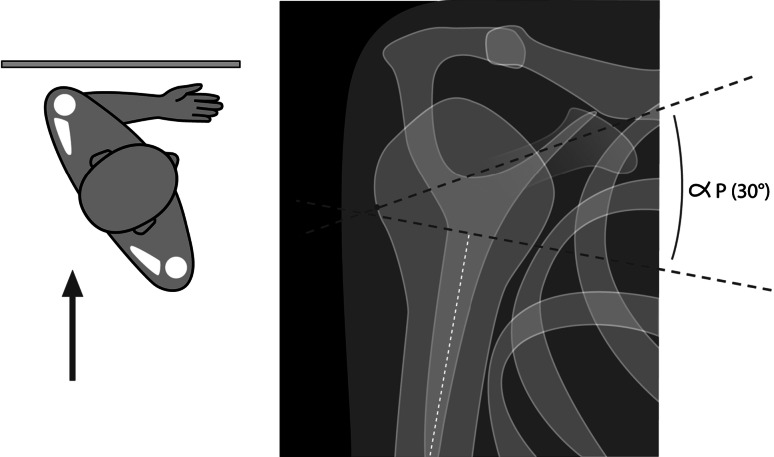
Lateral view

Results were assessed using the clinical Constant score (rough and weighted) [7], according to age and gender. Postoperative digital X-rays of the shoulder, which were carried out by the radiology department of each center (frontal and lateral view), were used to determine the quality of reduction. In this regard, the following angles were measured (Figs. 2, 3): alpha F of frontal tilt (looking for a valgus or residual varus on the frontal X-ray, with 45° considered as normal); alpha P of the sagittal tilt of the humeral head on the lateral view (assessing postoperative anterior or posterior tilt, with 30° considered as normal). In addition, the frontal displacement of the greater tuberosity in relation to the lateral edge of the articular surface was recorded by the three resident orthopedic surgeons. Finally, any complications related to these fractures were collected during the follow-up period, especially in cases involving second surgery.

**Fig. 4:**
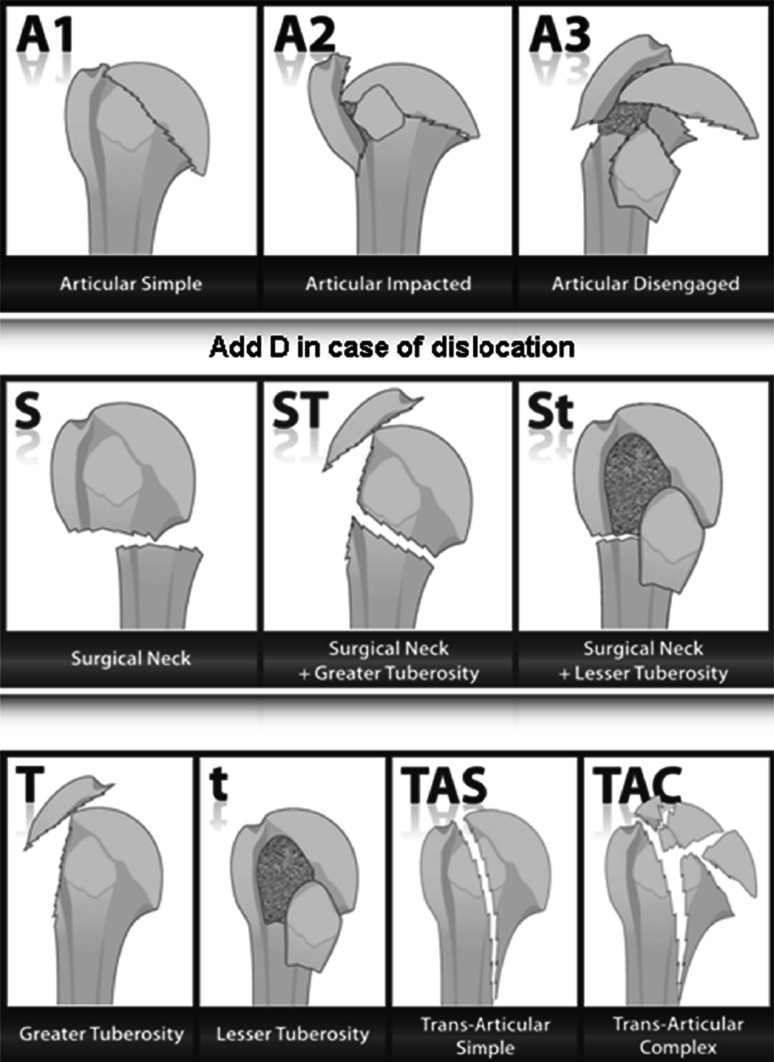
AST classification

**Fig. 5:**
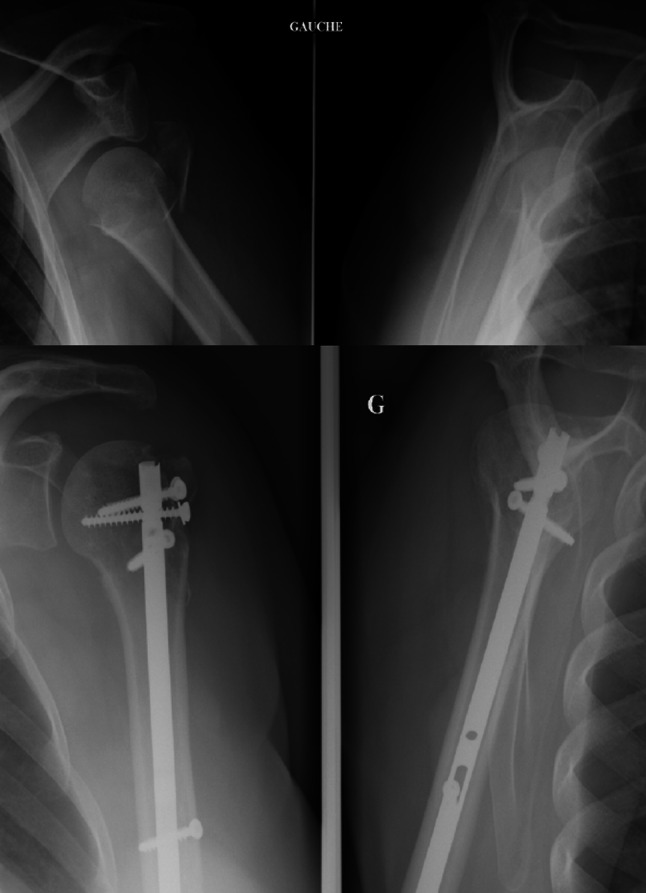
Dislocated valgus-impacted fracture (Neer 4-part, AST: A2D). Assembly with T4

**Fig. 6:**
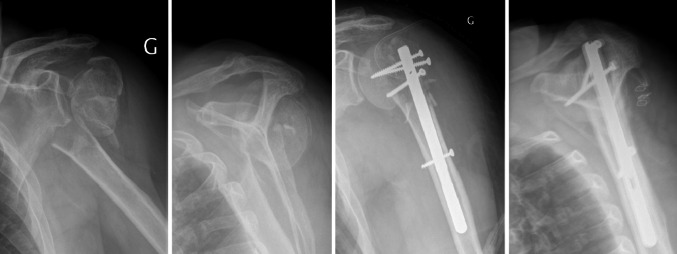
Complex articular disengaged fracture (Neer: 4-part, AST: A3). Assembly with T4

We used the Neer classification for fractures [8]. In addition, fractures were categorized according to the Articular Surgical neck Tuberosities (AST) classification [9] (Fig. 4), which is based on the articular or extra-articular characteristics of fractures (with or without the tuberosities) and differentiates articular (Fig. 5) valgus-impacted fractures (A2) from complex (Fig. 6), disengaged articular fractures (A3).

Descriptive statistics were computed. Quantitative variable were described as frequency and percentages and quantitative variables as mean, minimum, and maximum.

## Results

A total of 105 patients were eligible and enrolled in the study. Patient characteristics and fracture types are presented in Table 1. The majority of the subjects were women (74 %), and the mean age at the time of study inclusion was 69 years old (range 28–91 years). However, a total of 38 subjects were excluded due to the following: lost to follow-up (*n* = 20) or died prior to final follow-up (*n* = 18). Ultimately, 67 patients were available for final follow-up and data analysis. Thus, the final study population consisted of 51 women and 16 men with a mean age of 68 years old (range 28–86 years) (Table 2).

**Table 1:** Patient characteristics (age and gender) according to the different types of fractures

Type of fracture		Number		Mean age (in years)		Mean age of the groups (in years)
Neer	AST	Men	Women	Men	Women	
2-Part	S	9	23	62.5	69.3	67.4
3-Part	ST	11	32	73.6	71.1	71.7
4-Part simple	A2	2	11	54	65.2	62.7
4-Part complex	A3	3	12	77.3	72.3	73.3
Head splitting	TAC	2	–	59.5	–	59.5
Overall		27	78	67.9	69.9	69.4

**Table 2:** Surgical techniques according to the type of fracture

Type of fracture		Approach		Bone sutures	Crucifixion	Mean time of immobilization (in days)
Neer	AST	PC	AL			
2-Part	S	28	4	1	3	8
3-Part	ST	22	21	8	4	15
4-Part simple	A2	4	9	3	7	16
4-Part complex	A3	2	13	6	7	15
Head splitting	TAC	0	2	1	1	21

*PC* percutaneous, *AL* antero-lateral

### Complication rates

We found that 11 of the 67 patients (16 %) demonstrated postoperative complications, including device removal. Four patients (6 %) displayed secondary displacements, including two patients within the 4-part complex group. Both of these cases were associated with osteonecrosis of the humeral heads and required device removal and total shoulder arthroplasty (one anatomical prosthesis and one reverse prosthesis). In addition, we observed a secondary displacement of a surgical neck fracture, which required device removal and acromioplasty. Finally, there was one displacement within the 3-part group that caused a malunion without functional impairment.

Furthermore, three cases of postoperative complex regional pain syndrome were recorded. One of these cases occurred within the 2-part group and was completely resolvable, showing a weighted Constant score of 100 % at final follow-up. The remaining two cases were in the 4-part complex group. The first had secondary displacement of the fracture and associated osteonecrosis of the proximal end of the humerus and enjoyed regression of the symptoms following placement of a reverse prosthesis. The second case had a weighted Constant score of 51 % at final follow-up.

In addition, we observed five cases of osteonecrosis of the humeral heads in the 4-part complex group. Two were associated with secondary displacement of fragments. Three patients underwent secondary arthroplasty (two reverse prostheses and one anatomical). One patient had the Telegraph nail removed and was waiting for a replacement. The last case of osteonecrosis was well tolerated (weighted Constant score of 83 %).

Seven patients (10 %) received secondary treatments. Four patients underwent simple removal of the device (a screw in one case; screws and nail for the others). Three others displayed secondary shoulder arthroplasty (i.e., those patients with osteonecrosis discussed above).

Taken together, the majority of the complications were observed within the 4-part complex fractures group (6 of 11 patients; 67 % complication rate). On the other hand, two complications were seen in the 2-part group and three in the 3-part group, corresponding to rates of 10 and 9 %, respectively. Notably, no complications were associated with 4-part simple fractures.

### Clinical scores

In contrast to subjects who underwent simple removal of material, patients who needed follow-up surgery for arthroplasty were not clinically evaluated by Constant score (i.e., these represent failures). On the other hand, patients who underwent simple removal of their nail were evaluated. The mean duration for review was 38 months, and the mean rough Constant score, as evaluated from the entire set, was 64 (19–95). The weighted Constant score was 88 % (29–133).

An examination of results by type of fracture yielded a mean rough Constant score of 61 (19–84) for the 2-part group (surgical neck) and a mean weighted Constant score of 84 % (29–128). Three-part fractures (surgical neck with greater tuberosity fracture) had a mean rough Constant score of 69 (41–95) and a weighted score of 95 % (57–133). Here, we observed a mean rough Constant score of 62 (31–94) for the six 4-part simple fractures (valgus impacted) and a weighted score of 86 % (48–123). Finally, the mean rough score for the 4-part complex, disengaged, displaced joint fractures was 45 (33–53), whereas the weighted score was 67 % (51–82) (Table 4).

### X-ray evaluation

The study of postoperative reduction evaluated from X-rays showed a mean frontal angle of 44° (16°–71°) and a lateral angle of 36° (20°–74°) (Table 3). The mean frontal displacement of the greater tuberosity postoperatively was 1 mm (Table 4).

**Table 3:** Reduction criteria for postoperative X-ray images

Type of fracture		Alpha F	Alpha P
Neer	AST		
2-Part	S	44 (35–53)	34 (20–55)
3-Part	ST	44 (16–64)	37 (20–74)
4-Part simple	A2	46 (45–54)	33 (30–40)
4-Part complex	A3	44 (20–71)	37 (27–63)

**Table 4:** Functional scores and postoperative complications according to the type of fracture

Type of fracture		Mean rough constant	Mean weighted constant (%)	Postoperative complications
Neer	AST			
2-Part	S	61	84	10 % (2)
3-Part	ST	69	95	9 % (3)
4-Part simple	A2	62	86	0 %
4-Part complex	A3	45	67	67 % (6)
Overall (67 patients)		64	88	16 % (11)

## Discussion

In the present study, we assessed the radioclinical results and complications associated with a consecutive series of proximal humeral fractures treated with antegrade nailing using the Telegraph 4 system. We found that this technique showed good clinical results for extra-articular 2- and 3-part fractures with weighted Constant scores of 84 and 95 %, respectively. In addition, for these fracture types, we observed low rates of complications associated with this procedure (10 and 9 %, respectively).

**Fig. 7:**
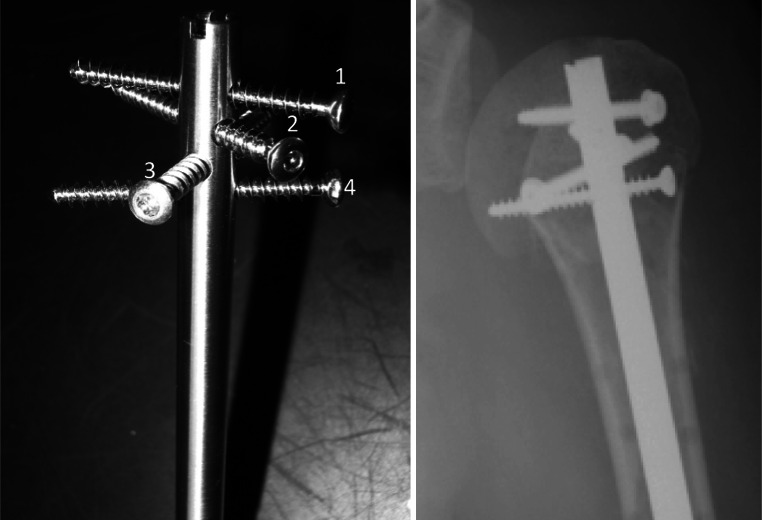
T4 nail, proximal end and X-ray

Overall, our evaluation of postoperative X-rays revealed a satisfactory reduction, with anatomical recovery of alpha F (44°) and alpha P (36°) angles and greater tuberosity. It should be noted that these results were similar regardless of the type of fracture. In comparison, using the initial Telegraph, Boughrebi et al. [10] found a mean alpha F angle of 38°, and Jayankura et al. [11] found six anatomical or sub-anatomical reductions out of 15 patients with 3- or 4-part Neer fractures. Thus, it seems that the addition of a fourth proximal screw and the polyaxial nature of the proximal locking seem to allow for better stabilization of the greater tuberosity, which offers significant advantages compared with previous versions of the device (Fig. 7). This may result in a satisfactory reduction and a stable osteosynthesis, thereby decreasing the risk of secondary displacement.

**Fig. 8:**
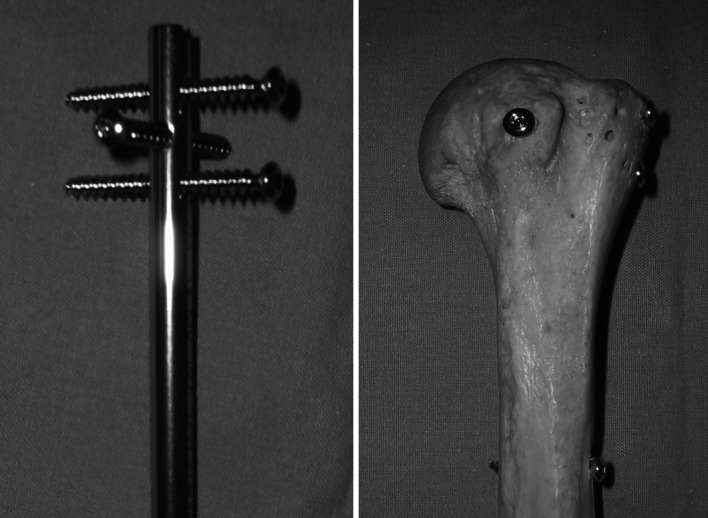
T1 nail, proximal end

In addition, we selected to use the weighted Constant score for evaluating clinical results in our study. This scoring system is more representative as a functional result, as it takes away male/female differences in muscular strength and muscular degeneration in the elderly. In this regard, we observed a mean Constant weighted score of 88 %, which is satisfactory from a general viewpoint, despite the existence of a significant rate of complications (16 %). However, Popescu et al. [12] found a weighted Constant score of 76 % in a set of 21 patients treated using the Stryker T2 nail, whereas Boughrebi et al. [10] found a score of 82.5 % with the initial Telegraph. Thus, the clinical results from our patient population appear to be satisfactory in comparison with the current data on proximal humeral nailing (Fig. 8).

Notably, the choice between freestanding nailing and osteosynthesis by locking plate for these fractures remains controversial. In fact, in 2009, Gradl et al. [13] examined a set of 152 patients with a diverse range of proximal humeral fractures and found no difference between locking plate osteosynthesis and Targon PH nailing (weighted Constant scores of 77 and 81 % along with complications rates of 28 and 22 %, respectively). Furthermore, Röderer et al. [14] and Königshausen et al. [15] described respective weighted Constant scores of 87 and 90.9 % along with complication rates of 30 and 23 % when analyzing two sets of osteosynthesis by locking plate (i.e., NCB and Suture plate). In this regard, our findings reveal a complication rate of 16 %, which is low compared with the literature and confirms the minimally invasive nature of modern nails.

So far, no study has been able to compare modern nails to osteosynthesis by locking plate. However, it seems that the general principle of nailing is subject to a lower rate of complications, as evidenced by the results of Zhu et al. [16], which showed no significant difference in terms of clinical results after 3 years (better for the plate after 1 year) between proximal humeral nailing and osteosynthesis by locking plate for 2-part fractures (S). Nevertheless, they reported a significantly lower rate of complications for nailing. Similarly, with respect to 3-part fractures (ST), Konrad et al. [17] found no clinical difference between nailing and locking plate.

Our analysis by fracture type also provided us with interesting information. In this respect, we classified fractures based on the Neer and AST systems. Notably, the latter allows differentiation between complex, disengaged articular fractures and more straightforward impacted articular fractures (most often valgus impacted). Indeed, the results of Telegraph 4 with regard to A2 valgus-impacted joint fractures appear equally satisfactory for 2-part (S) and 3-part (ST) fractures with a weighted Constant score of 86 %, reflecting a quality clinical recovery. In addition, there are no complications and particularly no necrosis or surgical follow-up. We must of course qualify this point in terms of the small sample size of the population (*n* = 6).

In contrast, we observed poor results for 4-part complex (A3), disengaged joint fractures, with a complication rate of 67 % (6 out of 9 patients reviewed). Indeed, there were two cases of reflex sympathetic dystrophy and two secondary displacements. The clinical results were also poor with a mean rough Constant score of 45, which was associated with a weighted score of 67 %. Despite the small sample of 4-part complex fractures in this study, it appears that treatment by nailing should be reserved for young patients. Thus, for this type of fracture, which is mostly found in osteoporotic subjects [2], prosthetic surgery remains to be the recommended indication.

There appears to be a significant difference in 4-part articular fractures depending on whether they are valgus impacted or disengaged, and this supports use of the AST classification [9]. Indeed, these two types of fractures are commonly classified together and can be linked to the majority of complications (especially osteonecrosis). Nevertheless, they seem to represent two well-differentiated entities. Therefore, nailing is a quality osteosynthesis solution for 4-part valgus-impacted joint fractures. Indeed, this concept has already been highlighted by Solberg et al. [18], who, when comparing results between osteosynthesis by plate and hemiarthroplasty in 3- and 4-part fractures, found a clinically significant benefit in favor of osteosynthesis for 3-part fractures and valgus-impacted fractures. In contrast, Reitman et al. [19] found a mean Constant score of 67 for complex comminuted fractures treated by reverse prosthesis. Also, Cai et al. [20] demonstrated a functional advantage in favor of hemiarthroplasty compared with osteosynthesis for 4-part displaced fractures in the elderly. However, our results could be criticized given the small number of cases, in particular with regard to the 4-part simple fractures.

From a technical point of view, it is essential to respect the entry point of the nail [4, 5] at the top of the head through the joint cartilage area in the diaphyseal axis to allow a reduction in quality and limit impact on the rotator cuff by passing through the muscle. This, combined with the short diameter of the Telegraph 4 and its screws, has the potential to result in better postoperative recovery. The percutaneous access used in 53 % of cases in this set is an advantage in elderly patients by allowing a rapid intervention, but should not be at the cost of a poorly positioned entry point or an incomplete reduction.

Notably, fractures of the proximal humerus represent a heterogeneous group of injuries. The high prevalence of these fractures in the elderly osteoporotic population must be taken into consideration because efficient osteosynthesis depends, among other things, upon the quality of the bone. Indeed, our cohort presented a mean age of 75 years and a strong female predominance, which is consistent with epidemiological data reported in the literature [1–3]. Thus, the findings we present here should be highly representative.

Finally, this study has several limitations. In particular, the small sample size may not have been sufficient for accurately assessing the efficacy of this technique for each of the fracture types. In addition, the follow-up may not have been adequate for assessing long-term complications that could arise from the procedure. Moreover, a large proportion of patients were lost to follow-up, which my have biased our results. However, since this study was conducted in injured elderly patients, it is difficult to avoid incomplete follow-up within this fragile population.

## Conclusions

Modern humerus intramedullary nails appear to be good options for the surgical treatment of proximal humerus fractures. The Telegraph 4 system offers a proximal locking option, which allows stabilization of the greater tuberosity, thereby reducing postoperative complications. In this respect, our clinical findings have yielded satisfactory outcomes with regard to extra-articular and valgus-impacted articular fractures, suggesting that antegrade nailing represents a valid treatment option for these indications. However, displaced, disengaged articular fractures seem to benefit from arthroplasty, which remains to be the recommended first-line treatment in elderly, osteoporotic patients. These findings contribute to a better understanding of the safety and efficacy of this therapeutic procedure and can facilitate the advancement of treatment options for humerus fractures.

## References

[1] Court-Brown CM, Caesar B (2006). Epidemiology of adult fractures: a review. Injury.

[2] Roux A, Decroocq L, El Batti S, Bonnevialle N, Moineau G, Trojani C, Boileau P, de Peretti F (2012). Epidemiology of proximal humerus fractures managed in a trauma center. Orthop Traumatol Surg Res.

[3] Court-Brown CM, Garg A, McQueen MM (2001). The epidemiology of proximal humeral fractures. Acta Orthop Scand.

[4] Bell JE, Leung BC, Spratt KF, Koval KJ, Weinstein JD, Goodman DC, Tosteson AN (2011). Trends and variation in incidence, surgical treatment, and repeat surgery of proximal humeral fractures in the elderly. J Bone Joint Surg Am.

[5] Cuny C, Pfeffer F, Irrazi M, Chammas M, Empereur F, Berrichi A, Metais P, Beau P (2002). A new locking nail for proximal humerus fractures: the Telegraph nail, technique and preliminary results. Rev Chir Orthop Reparatrice Appar Mot.

[6] Cuny C, Scarlat MM, Irrazi M, Beau P, Wenger V, Ionescu N, Berrichi A (2008). The Telegraph nail for proximal humeral fractures: a prospective four-year study. J Shoulder Elbow Surg.

[7] Constant CR, Murley AH (1987). A clinical method of functional assessment of the shoulder. Clin Orthop Relat Res.

[8] Neer CS (1970). Displaced proximal humeral fractures. I. Classification and evaluation. J Bone Joint Surg Am.

[9] Cuny C, Baumann C, Mayer J, Guignand D, Irrazi M, Berrichi A, Ionescu N, Guillemin F (2013). AST classification of proximal humeral fractures: introduction and interobserver reliability assessment. Eur J Orthop Surg Traumatol.

[10] Boughebri O, Havet E, Sanguina M, Daumas L, Jacob P, Zerkly B, Heissler P (2007). Treatment of proximal humeral fractures by Telegraph nail: prospective study of 34 cases. Rev Chir Orthop Reparatrice Appar Mot.

[11] Jayankura M, Phan DQ, Spinato L, Remy P, Cermak K, Schuind F (2011). Treatment of severe proximal humeral fractures by proximal nailing (Telegraph). A prospective preliminary study. Rev Med Brux.

[12] Popescu D, Fernandez-Valencia JA, Rios M, Cuñé J, Domingo A, Prat S (2009). Internal fixation of proximal humerus fractures using the T2-proximal humeral nail. Arch Orthop Trauma Surg.

[13] Gradl G, Dietze A, Kääb M, Hopfenmüller W, Mittlmeier T (2009). Is locking nailing of humeral head fractures superior to locking plate fixation?. Clin Orthop Relat Res.

[14] Röderer G, Erhardt J, Graf M, Kinzl L, Gebhard F (2010). Clinical results for minimally invasive locked plating of proximal humerus fractures. J Orthop Trauma.

[15] Königshausen M, Kübler L, Godry H, Citak M, Schildhauer TA, Seybold D (2012). Clinical outcome and complications using a polyaxial locking plate in the treatment of displaced proximal humerus fractures. A reliable system?. Injury.

[16] Zhu Y, Lu Y, Shen J, Zhang J, Jiang C (2011). Locking intramedullary nails and locking plates in the treatment of two-part proximal humeral surgical neck fractures: a prospective randomized trial with a minimum of three years of follow-up. J Bone Joint Surg Am.

[17] Konrad G, Audigé L, Lambert S, Hertel R, Südkamp NP (2012). Similar outcomes for nail versus plate fixation of three-part proximal humeral fractures. Clin Orthop Relat Res.

[18] Solberg BD, Moon CN, Franco DP, Paiement GD (2009). Surgical treatment of three and four-part proximal humeral fractures. J Bone Joint Surg Am.

[19] Reitman RD, Kerzhner E (2011). Reverse shoulder arthroplasty as treatment for comminuted proximal humeral fractures in elderly patients. Am J Orthop (Belle Mead NJ).

[20] Cai M, Tao K, Yang C, Li S (2012). Internal fixation versus shoulder hemiarthroplasty for displaced 4-part proximal humeral fractures in elderly patients. Orthopedics.

